# 3-Cyano­anilinium iodide monohydrate

**DOI:** 10.1107/S1600536810048294

**Published:** 2010-11-24

**Authors:** Jing Dai, Xin-Yuan Chen

**Affiliations:** a Ordered Matter Science Research Center, College of Chemistry and Chemical, Engineering, Southeast University, Nanjing 210096, People’s Republic of China

## Abstract

In the crystal structure of the title compound, C_7_H_7_N_2_^+^·I^−^·H_2_O, [C_7_H_7_N_2_^+^]_*n*_ chains extending along the *a*-axis direction are linked *via* N—H⋯N hydrogen bonds. The cations are further connected to the anions by N—H⋯I, N—H⋯O and O—H⋯I hydrogen bonds, leading to the formation of a sheet parallel to the *ac* plane. π–π inter­actions [centroid–centroid distance = 3.8378 (7) Å] link the sheets into a three-dimensional network.

## Related literature

For related structures, see: Oueslati *et al.* (2005 ▶); Messai *et al.* (2009 ▶). For applications of salts of amides as phase-transition dielectric materials, see: Fu *et al.* (2007 ▶, 2008 ▶, 2009 ▶); Fu & Xiong (2008 ▶).
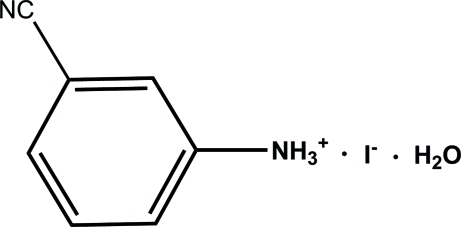


## Experimental

### 

#### Crystal data

C_7_H_7_N_2_^+^·I^−^·H_2_O
*M* *_r_* = 264.06
Monoclinic, 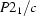

*a* = 8.0436 (16) Å
*b* = 16.603 (3) Å
*c* = 7.6746 (15) Å
β = 115.39 (3)°
*V* = 925.9 (3) Å^3^
*Z* = 4
Mo *K*α radiation
μ = 3.41 mm^−1^
*T* = 298 K
0.10 × 0.03 × 0.03 mm

#### Data collection

Rigaku Mercury2 diffractometer
Absorption correction: multi-scan (*CrystalClear*; Rigaku, 2005 ▶) *T*_min_ = 0.910, *T*_max_ = 1.000
9455 measured reflections
2117 independent reflections
1978 reflections with *I* > 2σ(*I*)
*R*_int_ = 0.041

#### Refinement

*R*[*F* ^2^ > 2σ(*F* ^2^)] = 0.029
*wR*(*F* ^2^) = 0.061
*S* = 1.21
2117 reflections
102 parameters
H-atom parameters constrained
Δρ_max_ = 0.72 e Å^−3^
Δρ_min_ = −0.89 e Å^−3^

### 

Data collection: *CrystalClear* (Rigaku, 2005 ▶); cell refinement: *CrystalClear*; data reduction: *CrystalClear*; program(s) used to solve structure: *SHELXS97* (Sheldrick, 2008 ▶); program(s) used to refine structure: *SHELXL97* (Sheldrick, 2008 ▶); molecular graphics: *SHELXTL* (Sheldrick, 2008 ▶); software used to prepare material for publication: *SHELXTL*.

## Supplementary Material

Crystal structure: contains datablocks I, global. DOI: 10.1107/S1600536810048294/vm2061sup1.cif

Structure factors: contains datablocks I. DOI: 10.1107/S1600536810048294/vm2061Isup2.hkl

Additional supplementary materials:  crystallographic information; 3D view; checkCIF report

## Figures and Tables

**Table 1:** Hydrogen-bond geometry (Å, °)

*D*—H⋯*A*	*D*—H	H⋯*A*	*D*⋯*A*	*D*—H⋯*A*
N1—H1*C*⋯N2^i^	0.89	2.11	2.991 (4)	169
N1—H1*A*⋯O1*W*^ii^	0.89	1.98	2.850 (4)	164
N1—H1*B*⋯I1^iii^	0.89	2.60	3.487 (3)	171
O1*W*—H1*WB*⋯I1^ii^	0.93	2.75	3.635 (3)	159
O1*W*—H1*WA*⋯I1	0.96	2.65	3.576 (3)	162

Symmetry codes: (i) 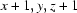; (ii) 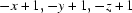; (iii) 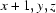.

**Fig. 1.**
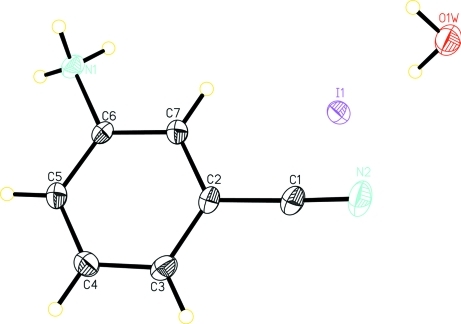
A view of the title compound with the atomic numbering scheme. Displacement ellipsoids were drawn at the 30% probability level.

**Fig. 2.**
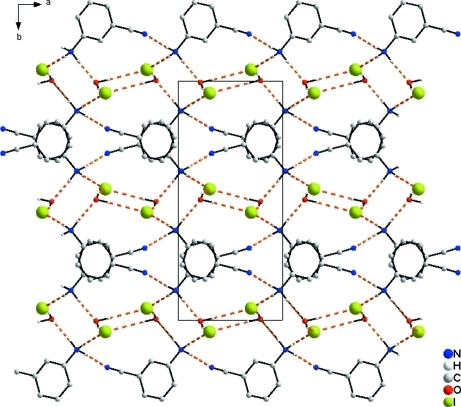
The crystal packing of the title compound, showing the two-dimensional hydrogen-bonded network parallel to the ac plane. H atoms not involved in hydrogen bonding (dashed line) have been omitted for clarity.

**Fig. 3.**
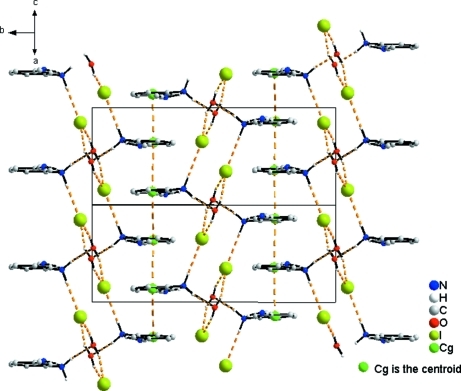
The crystal packing of the title compound showing the π-π interactions linking the hydrogen-bonded network into a three-dimensional network.

## supplementary crystallographic information

### Comment

Salts of amides attracted much attention as phase transition dielectric materials for applications in micro-electronics and memory storage (Fu *et al.* 2007; Fu & Xiong 2008; Fu *et al.* 2008; Fu *et al.* 2009). With the purpose of obtaining phase transition crystals of 3-aminobenzonitrile salts, its interaction with various acids has been studied and we have elaborated a series of new materials with this organic molecule. In this paper, we describe the crystal structure of the title compound, 3-cyanoanilinium iodine monohydrate.

The asymmetric unit is composed of a iodine anion, a 3-cyanoanilinium cation and a water molecule (Fig.1). The geometric parameters of the title compound agree well with reported similar structures (Oueslati *et al.*, 2005; Messai *et al.*, 2009). The cation is almost planar (r.m.s. deviation 0.0097 Å for best plane through all non-H atoms of cation). Moreover, the C—NH_3_ (1.459 (2)Å) and C≡N (1.132 (3) Å) distances in the 3-cyanoanilinium cation are almost equal with respect to the C—NH_3_ (1.457 (4) Å) and C≡ N (1.137 (4) Å) observed in the crystal structure of 2-cyanoanilinium chloride (Oueslati *et al.*, 2005).

The cations are surrounded by the anions and water molecules *via* hydrogen bonds which play an important role in stabilizing the crystal structure. In the crystal structure, all the amine group H atoms are involved in N—H···I, N—H···O and N—H···N hydrogen bonds with N···I, N···O and N···N distances of 3.487 (3) Å, 2.850 (4)Å and 2.991 (4) Å. These hydrogen bonds link the ionic units into a two-dimensional graph-set motif parallel to the *ac* plane (Table 1, Fig. 2). Furthermore, π–π interactions [Cg(1)···Cg(1)^i^ = 3.8378 (7) Å; Cg(1) is centroid of ring C2 - C7; symmetry operation: (i) *x*, 1/2 - *y*, 1/2 + *z* ] link the sheets into a three-dimensional network (Fig.3).

### Experimental

The commercial available 3-aminobenzonitrile (3 mmol, 324 mg) was dissolved in water/HI (50:1 *v*/*v*) solution. The solvent was slowly evaporated in air affording colourless block-shaped crystals of the title compound suitable for X-ray analysis.

While permittivity measurements show that there is no phase transition within the temperature range (from 100 K to 400 K), the permittivity is 6.8 at 1 MHz at room temperature.

### Refinement

All H atoms attached to C atoms were fixed geometrically and treated as riding with C–H = 0.93 Å, with *U*_iso_(H) = 1.2Ueq(C). The NH_3_^+^ H atoms were calculated geometrically and were refined using a riding model with N—H = 0.89 Å, with *U*_iso_(H) = 1.5*U*_eq_(N). A rotating-group model was used for the –NH_3_ group. H atoms of water molecule were located in difference Fourier maps and freely refined. In the last stage of refinement they were treated as riding on the O atom, with *U*_iso_(H) = 1.5*U**_eq_*(O).

### Crystal data

**Table:**

C_7_H_7_N_2_^+^·I^−^·H_2_O	*F*(000) = 504
*M**_r_* = 264.06	*D*_x_ = 1.894 Mg m^−^^3^
Monoclinic, *P*2_1_/*c*	Mo *K*α radiation, λ = 0.71073 Å
Hall symbol: -P 2ybc	Cell parameters from 2117 reflections
*a* = 8.0436 (16) Å	θ = 3.1–27.5°
*b* = 16.603 (3) Å	µ = 3.41 mm^−^^1^
*c* = 7.6746 (15) Å	*T* = 298 K
β = 115.39 (3)°	Block, colorless
*V* = 925.9 (3) Å^3^	0.10 × 0.03 × 0.03 mm
*Z* = 4	

### Data collection

**Table:**

Rigaku Mercury2 diffractometer	2117 independent reflections
Radiation source: fine-focus sealed tube	1978 reflections with *I* > 2σ(*I*)
graphite	*R*_int_ = 0.041
Detector resolution: 13.6612 pixels mm^-1^	θ_max_ = 27.5°, θ_min_ = 3.1°
CCD profile fitting scans	*h* = −10→10
Absorption correction: multi-scan (*CrystalClear*; Rigaku, 2005)	*k* = −21→21
*T*_min_ = 0.910, *T*_max_ = 1.000	*l* = −9→9
9455 measured reflections	

### Refinement

**Table:**

Refinement on *F*^2^	Secondary atom site location: difference Fourier map
Least-squares matrix: full	Hydrogen site location: inferred from neighbouring sites
*R*[*F*^2^ > 2σ(*F*^2^)] = 0.029	H-atom parameters constrained
*wR*(*F*^2^) = 0.061	*w* = 1/[σ^2^(*F*_o_^2^) + (0.0103*P*)^2^ + 0.9565*P*] where *P* = (*F*_o_^2^ + 2*F*_c_^2^)/3
*S* = 1.21	(Δ/σ)_max_ < 0.001
2117 reflections	Δρ_max_ = 0.72 e Å^−^^3^
102 parameters	Δρ_min_ = −0.89 e Å^−^^3^
0 restraints	Extinction correction: *SHELXL97* (Sheldrick, 2008), Fc^*^=kFc[1+0.001xFc^2^λ^3^/sin(2θ)]^-1/4^
Primary atom site location: structure-invariant direct methods	Extinction coefficient: 0.0824 (16)

### Special details

**Table:**

Geometry. All e.s.d.'s (except the e.s.d. in the dihedral angle between two l.s. planes) are estimated using the full covariance matrix. The cell e.s.d.'s are taken into account individually in the estimation of e.s.d.'s in distances, angles and torsion angles; correlations between e.s.d.'s in cell parameters are only used when they are defined by crystal symmetry. An approximate (isotropic) treatment of cell e.s.d.'s is used for estimating e.s.d.'s involving l.s. planes.
Refinement. Refinement of *F*^2^ against ALL reflections. The weighted *R*-factor *wR* and goodness of fit *S* are based on *F*^2^, conventional *R*-factors *R* are based on *F*, with *F* set to zero for negative *F*^2^. The threshold expression of *F*^2^ > σ(*F*^2^) is used only for calculating *R*-factors(gt) *etc*. and is not relevant to the choice of reflections for refinement. *R*-factors based on *F*^2^ are statistically about twice as large as those based on *F*, and *R*- factors based on ALL data will be even larger.

### Fractional atomic coordinates and isotropic or equivalent isotropic displacement parameters (Å^2^)

**Table:**

	*x*	*y*	*z*	*U*_iso_*/*U*_eq_	
N1	1.0275 (3)	0.37559 (16)	0.8592 (4)	0.0332 (6)	
H1A	0.9606	0.4146	0.8775	0.050*	
H1B	1.0861	0.3949	0.7927	0.050*	
H1C	1.1093	0.3575	0.9730	0.050*	
N2	0.3209 (4)	0.3013 (2)	0.2122 (4)	0.0483 (8)	
C1	0.4542 (4)	0.2842 (2)	0.3388 (4)	0.0350 (7)	
C2	0.6270 (4)	0.26503 (19)	0.4998 (4)	0.0288 (6)	
C3	0.6773 (4)	0.18539 (19)	0.5508 (5)	0.0343 (7)	
H3	0.5995	0.1436	0.4829	0.041*	
C4	0.8443 (4)	0.1692 (2)	0.7037 (5)	0.0365 (7)	
H4	0.8788	0.1161	0.7394	0.044*	
C5	0.9614 (4)	0.23118 (18)	0.8048 (4)	0.0306 (6)	
H5	1.0747	0.2200	0.9065	0.037*	
C6	0.9070 (4)	0.30971 (17)	0.7520 (4)	0.0257 (6)	
C7	0.7422 (4)	0.32826 (19)	0.6002 (4)	0.0294 (6)	
H7	0.7085	0.3815	0.5653	0.035*	
O1W	0.2139 (3)	0.49557 (16)	0.1596 (4)	0.0488 (6)	
H1WA	0.2348	0.4707	0.2806	0.073*	
H1WB	0.3325	0.5071	0.1742	0.073*	
I1	0.30166 (3)	0.451061 (14)	0.64728 (3)	0.04440 (13)	

### Atomic displacement parameters (Å^2^)

**Table:**

	*U*^11^	*U*^22^	*U*^33^	*U*^12^	*U*^13^	*U*^23^
N1	0.0275 (13)	0.0362 (14)	0.0302 (13)	−0.0081 (10)	0.0067 (10)	−0.0028 (11)
N2	0.0317 (15)	0.060 (2)	0.0379 (15)	0.0013 (13)	0.0001 (13)	−0.0064 (14)
C1	0.0271 (15)	0.0425 (18)	0.0300 (16)	−0.0034 (13)	0.0073 (13)	−0.0073 (13)
C2	0.0214 (13)	0.0374 (16)	0.0244 (14)	−0.0004 (12)	0.0068 (11)	−0.0034 (12)
C3	0.0298 (15)	0.0344 (16)	0.0380 (16)	−0.0077 (12)	0.0139 (13)	−0.0100 (13)
C4	0.0361 (16)	0.0293 (16)	0.0391 (17)	0.0031 (13)	0.0116 (14)	0.0000 (13)
C5	0.0244 (14)	0.0367 (16)	0.0277 (14)	0.0035 (12)	0.0083 (12)	0.0028 (12)
C6	0.0217 (13)	0.0311 (15)	0.0231 (13)	−0.0054 (11)	0.0086 (11)	−0.0033 (11)
C7	0.0258 (14)	0.0307 (15)	0.0278 (14)	0.0013 (11)	0.0079 (11)	0.0014 (11)
O1W	0.0377 (13)	0.0442 (14)	0.0516 (15)	−0.0006 (11)	0.0070 (11)	−0.0055 (11)
I1	0.03749 (16)	0.04184 (17)	0.05167 (18)	0.00206 (9)	0.01702 (12)	0.01218 (10)

### Geometric parameters (Å, °)

**Table:**

N1—C6	1.459 (4)	C3—H3	0.9300
N1—H1A	0.8900	C4—C5	1.386 (4)
N1—H1B	0.8900	C4—H4	0.9300
N1—H1C	0.8900	C5—C6	1.380 (4)
N2—C1	1.132 (4)	C5—H5	0.9300
C1—C2	1.444 (4)	C6—C7	1.373 (4)
C2—C3	1.389 (4)	C7—H7	0.9300
C2—C7	1.394 (4)	O1W—H1WA	0.9626
C3—C4	1.380 (4)	O1W—H1WB	0.9316
			
C6—N1—H1A	109.5	C3—C4—C5	120.8 (3)
C6—N1—H1B	109.5	C3—C4—H4	119.6
H1A—N1—H1B	109.5	C5—C4—H4	119.6
C6—N1—H1C	109.5	C6—C5—C4	118.9 (3)
H1A—N1—H1C	109.5	C6—C5—H5	120.5
H1B—N1—H1C	109.5	C4—C5—H5	120.5
N2—C1—C2	178.0 (4)	C7—C6—C5	122.0 (3)
C3—C2—C7	121.1 (3)	C7—C6—N1	118.5 (3)
C3—C2—C1	120.5 (3)	C5—C6—N1	119.5 (3)
C7—C2—C1	118.3 (3)	C6—C7—C2	118.1 (3)
C4—C3—C2	119.0 (3)	C6—C7—H7	120.9
C4—C3—H3	120.5	C2—C7—H7	120.9
C2—C3—H3	120.5	H1WA—O1W—H1WB	103.1

### Hydrogen-bond geometry (Å, °)

**Table:**

*D*—H···*A*	*D*—H	H···*A*	*D*···*A*	*D*—H···*A*
N1—H1C···N2^i^	0.89	2.11	2.991 (4)	169
N1—H1A···O1W^ii^	0.89	1.98	2.850 (4)	164
N1—H1B···I1^iii^	0.89	2.60	3.487 (3)	171
O1W—H1WB···I1^ii^	0.93	2.75	3.635 (3)	159
O1W—H1WA···I1	0.96	2.65	3.576 (3)	162

Symmetry codes: (i) *x*+1, *y*, *z*+1; (ii) −*x*+1, −*y*+1, −*z*+1; (iii) *x*+1, *y*, *z*.
